# The evaluation of the MMP-2/TIMP-1 ratio in peptic ulcer and its association with refractory helicobacter pylori infection

**DOI:** 10.1186/s12876-023-02923-z

**Published:** 2023-08-21

**Authors:** Mohammad Negaresh, Elham Safarzadeh, Nasrin Fouladi, Somaieh Matin, Sanaz Pourfarzi

**Affiliations:** 1 Department of Internal Medicine, Faculty of Medicine, Ardabil University of Medical Sciences, Ardabil, Iran; 2 School of Medicine and Allied Medical Sciences, Ardabil University of Medical Sciences, Ardabil, Iran; 3 Social Determinants of Health Research Center, Ardabil University of Medical Sciences, Ardabil, Iran; 4 Gastroenterology and Hepatology, Department of Internal Medicine, School of Medicine, Digestive Diseases Research Center, Ardabil University of Medical Sciences, Ardabil, Iran; 5 Students Research Committee, School of Medicine, Ardabil University of Medical Sciences, Ardabil, Iran

**Keywords:** MMP-2, TIMP-1, Peptic ulcer, Helicobacter pylori infection

## Abstract

**Background:**

Helicobacter pylori (*H. pylori*) is one of the leading causes of peptic ulcers, and its treatment is a worldwide challenge. Matrix metalloproteinases and their inhibitors influence the development and healing of peptic ulcers. This study aimed to evaluate the ratios of matrix metalloproteinase-2 (MMP-2) to tissue inhibitor of metalloproteinase-1 (TIMP-1) in patients with peptic ulcers that are sensitive or resistant to *H. pylori* treatment and compare them with healthy individuals.

**Methods:**

In this study, 95 patients were included and divided into two groups sensitive (41 patients) and resistant to treatment (54 patients). The results were compared with a control group of 20 participants with normal endoscopy and *H. pylori-negative*. After obtaining written informed consent, five ml of venous blood was taken to determine their serum MMP-2 and TIMP-1 levels using an enzyme-linked immunosorbent assay.

**Results:**

In patients with *H. pylori*-induced peptic ulcers, the MMP-2/TIMP-1 ratio was significantly higher than the healthy controls (*P* < 0.05). MMP-2 level was associated with patients’ response to treatment (*P* < 0.05). The MMP-2/TIMP-1 ratio was higher in patients with simultaneous gastric and duodenal ulcers (*P* < 0.05).

**Conclusion:**

It seems that peptic ulcer disease caused by infection with *H. pylori* increases the MMP-2/TIMP-1 ratio in patients with peptic ulcers. However, it might not be a good predictor of refractory *H. pylori*-induced peptic ulcer disease.

## Background

Peptic ulcer disease (PUD) is a prevalent condition in the digestive system which usually occurs in the stomach or duodenum due to the damage caused by peptic acid [1]. Helicobacter pylori (*H. pylori*) and non-steroidal anti-inflammatory drugs (NSAIDs) are the leading cause of PUD. However, only a limited number of *H.pylori*-positive patients or NSAID consumers become afflicted with PUD [2, 3]. Diagnostic tests for detecting active *H. pylori* infection include endoscopy, urea breath test, and stool antigen test. A stool antigen test is usually used to primarily detect *H.pylori* infection and confirm its eradication [4, 5]. *H.pylori* infection is common in Iran, and its prevalence is between 36 and 90% in different geographic areas [6].

Due to the increase in antibiotic resistance and failure of treatment in almost 20% of the patient, the first attempt to eradicate *H. pylori* has become a challenge. It is essential to perform a confirmation test to verify that *H. pylori* has been completely eradicated after the eradication therapy. The stool antigen test is a widely used and effective tool for this purpose [7].

Matrix metalloproteinases (MMPs) are zinc-dependent proteolytic enzymes that degrade the basement membrane (BM) and extracellular matrix (ECM) components [8]. Tissue inhibitors of metalloproteinases (TIMPs) are endogenous and naturally occurring inhibitors of MMPs, inhibiting their actions by creating noncovalent complexes. Four members of TIMPs, TIMP-1, -2, -3, and -4, have been characterized. The balance between MMPs and TIMPs is responsible for regulating the turnover of the extracellular matrix and maintaining tissue homeostasis. Any changes in this balance can lead to the development of various diseases. TIMP-1 is a potent inhibitor of several MMPs and also plays a crucial role in controlling different biological processes, including cell growth, proliferation, and apoptosis, by binding to undisclosed receptors [9].

If an individual contracts H. pylori, it will ultimately result in the production of MMPs. This leads to a state of chronic inflammation, causing severe harm to the mucosal lining. Furthermore, it creates an environment that makes it easier for bacteria, immune cells, and stroma to connect with the epithelium, thereby exacerbating the situation [10].

Several studies have examined MMP levels in tissue and serum samples of patients with H. pylori infection. In the tissue sample, MMP types 2, 7, 8, and 9, as well as MT1-MMP, were found to be elevated, but research on TIMP-1 and TIMP-2 has yielded conflicting results [11–13]. When using the ELISA method to examine the serum of adults with PUD caused by H. pylori and comparing it to healthy individuals, the results indicated that the levels of MMP-8 and -9 increased while the level of MMP-7 did not change. Additionally, the levels of MMP-2 and TIMP-1 decreased [14].

There have been conflicting reports about the levels of MMP-2 and TIMP-1 in patients with H. pylori infection. While some studies have shown a decrease in these levels, others have found the opposite [14, 15]. This has made establishing a clear association between H. pylori infection and MMP-2 and TIMP-1 levels difficult. Additionally, there has been no research on the link between MMPs/TIMPs and resistant H. pylori infection. This study aims to investigate the MMP-2/TIMP-1 ratio in patients with PUD and its relationship with refractory H. pylori infection. By measuring this ratio before starting treatment, it may be possible to predict the likelihood of resistance and prescribe a more effective treatment plan. This approach will be more cost-effective and reduce the risk of antibiotic resistance while improving patient tolerance.

## Methods

### Study design

This analytical cross-sectional study investigated patients referring to the Gastroenterology Clinic of Imam Khomeini Hospital of Ardabil, Iran. A checklist of demographic information, including age, sex, etc., and endoscopy results were filled out for each patient.

Then they underwent standard quadruple therapy for *H. pylori* infection with 14 days of omeprazole (20 mg twice a day), metronidazole (500 mg twice a day), clarithromycin (500 mg twice a day), and bismuth subsalicylate (two tablets, four times a day). A stool antigen test was performed 8–12 weeks after therapy to confirm *H. pylori* eradication.

**Fig. 1:**
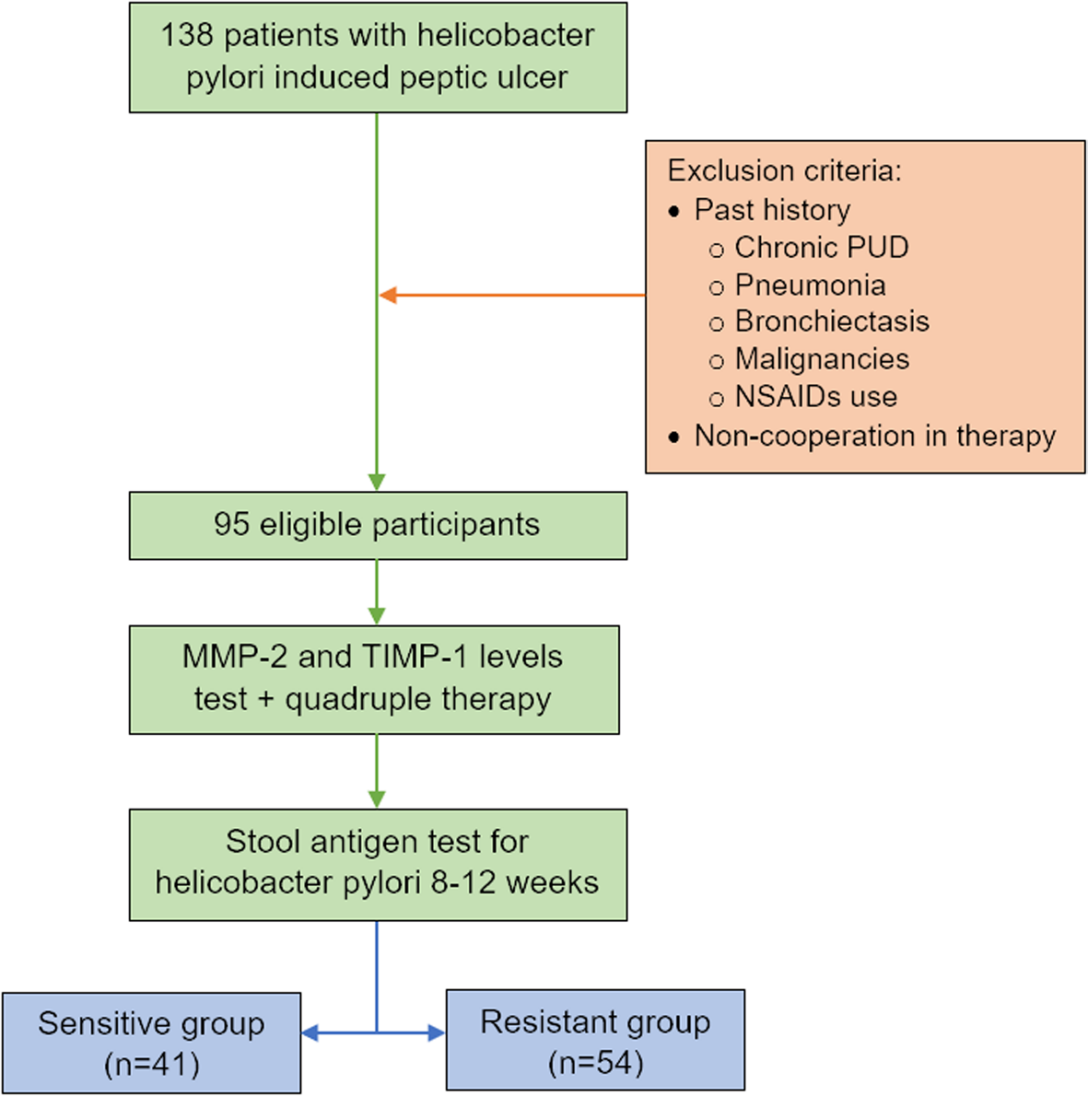
The study design flowchart

To evaluate the serum MMP-2 and TIMP-1 levels, 5 ml of blood sample was taken before treatment began. After keeping the blood samples for 20 min at room temperature, samples were centrifuged at 2500–3000 rpm for 10–15 min to separate the serum. The samples were then transferred to a refrigerator and stored at -80 °C until use. Finally, the samples were analyzed for serum MMP-2 and TIMP-1 levels using ELISA kits (ZellBio®, Germany) according to the manufacturer’s protocol. In Fig. 1, the flowchart of the study design is illustrated.

### Samples preparation

The patients who entered the study were diagnosed with PUD and *H. pylori* infection based on their endoscopy and biopsy results. We excluded participants with chronic PUD, pneumonia, bronchiectasis, and a history of using NSAIDs or having malignancies from the study as these conditions have the potential to affect the MMP-2 or TIMP-1 levels and could introduce bias to the results [16–19]. Ninety-five patients participated in the study and were divided into two groups. The resistant group consisted of 54 patients who still tested positive, while the sensitive group had 41 patients who tested negative. Additionally, a control group was included, which consisted of 20 healthy individuals with normal endoscopy and negative H. pylori test.

### Ethical approval

The present study was approved by the Research Ethics Committee of the University of Medical Sciences with the approval code of IR.ARUMS.REC.1399.081. Before entering the study, written informed consent was obtained from each patient.

### Statistical analyses

The obtained data were coded and fed into SPSS Software, Version 26. The Chi-square test was used to determine the relationship between the treatment response and variables. Also, to evaluate the relationship between variables and serum levels of MMP-2, TIMP-1, and their ratios, the one-way ANOVA and independent t-test were used. The significance level was set at 0.05 for all tests.

## Results

### Demographic features and endoscopy findings of the enrolled population

The study examined a total of 115 participants, including 95 patients with *H. pylori*-positive PUD and 20 healthy individuals. Of these participants, 70 were male, and 45 were female, with an average age of 52.23 ± 9.91 years. The use of alcohol among the three groups showed significant differences (*P* < 0.05), according to the Chi-Square test, but the endoscopic findings did not show any significant results (*P* = 0.65) (Table 1).

**Table 1:** The distribution of demographic features and endoscopy findings in the study groups

Variables	Patients		Control	*P*-value
	**Sensitive (*n* = 41)**	**Resistant (*n* = 54)**		
**Gender**				
Male	23 (20%)	35 (30.4%)	12 (10.4%)	0.68
Female	18 (15.7%)	19 (16.5%)	8 (7%)	
**Age (Years)**				
< 40	2 (1.7%)	6 (5.2%)	2 (1.7%)	0.44
40 -54	17 (14.8%)	29 (25.2%)	10 (8.7%)	
55 -70	22 (19.1%)	19 (16.5%)	8 (7%)	
**Alcohol**				
Yes	6 (5.2%)	1 (0.9%)	4 (3.5%)	**0.02**
No	35 (30.4%)	53 (46.1%)	16 (13.9%)	
**Smoking**				
Yes	8 (7%)	11 (9.6%)	0 (0%)	0.09
No	33 (28.7%)	43 (37.4%)	20 (17.4%)	
**Hookah**				
Yes	3 (2.6%)	6 (5.2%)	0 (0%)	0.28
No	38 (33%)	48 (41.7%)	20 (17.4%)	
**Endoscopic findings**				
Gastropathy	17 (17.9%)	22 (23.1%)	-	0.65
Bulbopathy	7 (7.4%)	6 (6.3%)	-	
Gastrobulbopathy	17 (17.9%)	26 (27.4%)	-	

The results are achieved using Chi-Square Test

### Comparison of MMP-2 and TIMP-1 ratios with demographic features and endoscopy findings

The levels of MMP-2, TIMP-1, and their ratio were found to be related to alcohol consumption and smoking, with statistical significance (*P* < 0.05). Patients with a smoking history and those without a history of alcohol consumption had higher levels of MMP-2. Smoking also caused a significant increase in the MMP-2/TIMP-1 ratio (*P* = 0.01). Endoscopic findings showed a significant association with MMP-2, TIMP-1, and their ratio (*P* < 0.05), with the highest levels of MMP-2 and MMP-2/TIMP-1 ratio and the lowest levels of TIMP-1, observed in patients with PUD simultaneously present in the gastric and duodenal regions (Table 2).

**Table 2:** Analysis of mean MMP-2 and TIMP-1 levels and their relationship with demographic features and endoscopy findings

Variables	MMP-2 (ng/ml)	*P*-value	TIMP-1 (ng/ml)	*P*-value	MMP-2/TIMP-1 ratios	*P*-value
**Age (Years)**						
< *40*	740.90 ± 250.34	0.54	204.81 ± 80.42	0.76	3.34 ± 1.17	0.40
*40—54*	632.12 ± 274.25		184.77 ± 70.50		3.77 ± 2.01	
*55 -70*	648.27 ± 285.99		189.56 ± 89.70		4.24 ± 3.01	
**Alcohol**						
*Yes*	449.27 ± 265.70	**0.02**	182.27 ± 75.44	0.77	2.70 ± 1.97	0.052
*No*	669.53 ± 280.07		189.23 ± 80.29		4.06 ± 2.46	
**Smoking**						
*Yes*	811.84 ± 234.72	**0.003**	194.89 ± 73.42	0.68	5.06 ± 2.08	**0.01**
*No*	616.13 ± 284.11		187.31 ± 81.1		3.71 ± 2.45	
**Hookah**						
*Yes*	820.11 ± 265.43	0.07	200.72 ± 71.94	0.61	5.19 ± 2.47	0.14
*No*	633.89 ± 283.12		187.53 ± 80.39		3.83 ± 2.42	
**Endoscopic findings**						
*Gastropathy*	695.25 ± 215.56	** < 0.001**	195.46 ± 62.70	**0.04**	3.78 ± 1.64	**0.002**
*Bulbopathy*	642.67 ± 205.42		229.10 ± 92.56		3.83 ± 2.43	
*Gastrobulbopathy*	863.50 ± 202.06		188.63 ± 71.05		5.56 ± 3.04	
*Control*	633 ± 155.49		243.77 ± 92.44		3.33 ± 1.65	

*MMP* Matrix metalloproteinase, *TIMP* Tissue inhibitor of matrix metalloproteinase. The results are achieved using one-way ANOVA

### Comparison of MMP-2/TIMP-1 ratio in the study groups

**Fig. 2:**
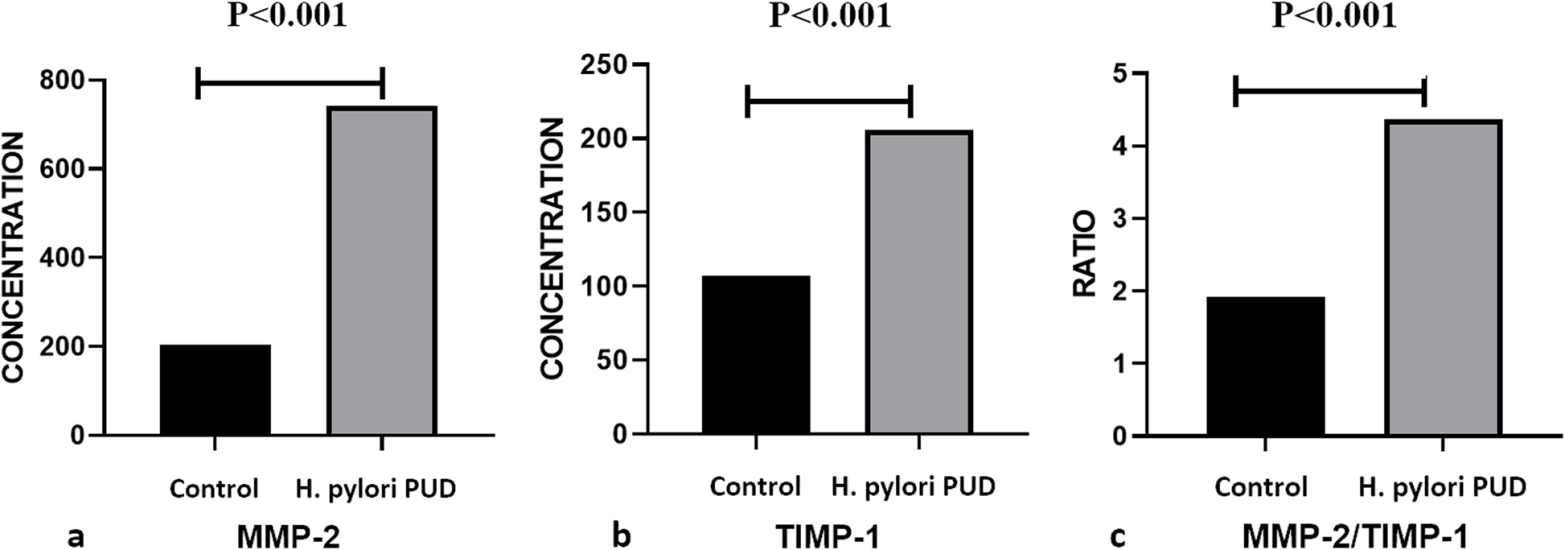
The comparison of mean serum levels of MMP-2, TIMP-1, and MMP-2/TIMP-1 ratios between patients with H. pylori induced peptic ulcer and control groups

The mean MMP-2 level was 741.93 ± 218.16 ng/ml in the *H. pylori-induced* PUD group and 204.50 ± 33.80 ng/ml in the control group. The mean TIMP-1 level for these two groups was 205.67 ± 77.18 ng/ml and 107.31 ± 9.27 ng/ml, respectively. Furthermore, the mean MMP-2/TMP-1 ratio in these two groups was 4.36 ± 2.48 ng/ml and 1.92 ± 0.39 ng/ml, respectively. The results of the analyses indicated that the patient and control groups were significantly different in terms of mean levels of MMP-2, TIMP-1, and MMP-2/TIMP-1 ratio (*P* < 0.001) (Fig. 2).

**Fig. 3:**
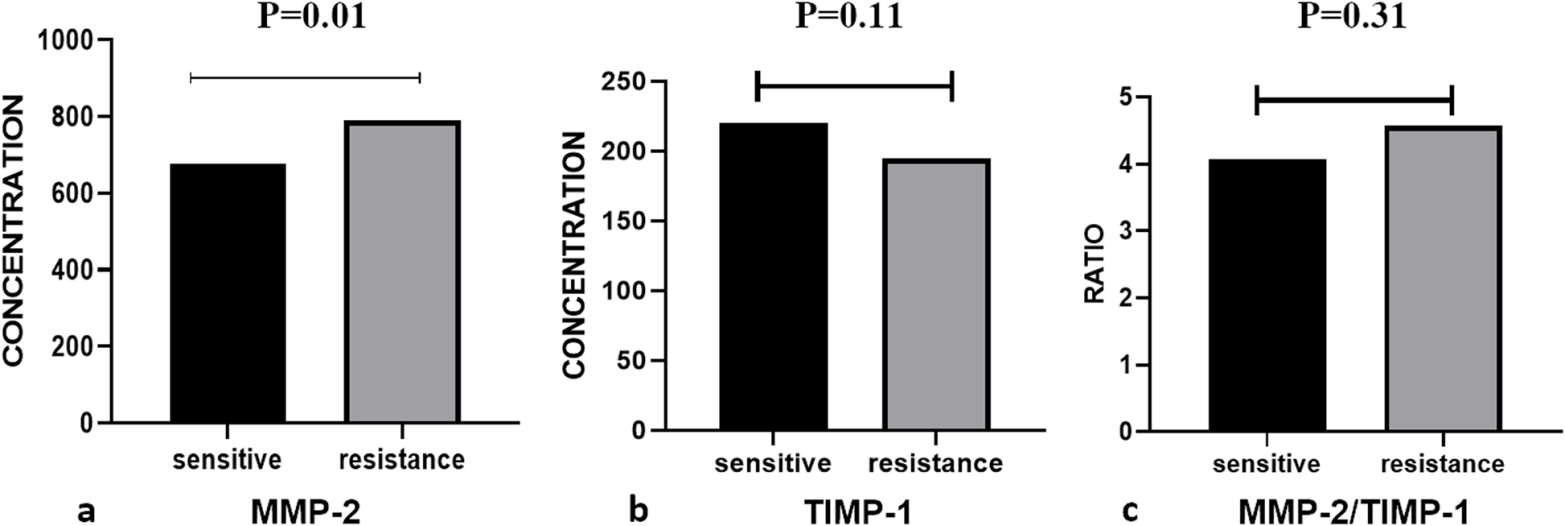
The comparison of mean serum levels of MMP-2, TIMP-1, and their ratios between H. pylori treatment sensitive and resistant patients

The mean MMP-2 level in the sensitive group was 677.34 ± 177.28 ng/ml, and in the resistant group was 790.96 ± 234.56 ng/ml. The mean TIMP-1 levels for these two groups were 220.49 ± 88.99 ng/ml and 194.42 ± 65.49 ng/ml, respectively. Also, the mean MMP-2/TIMP-1 ratios in the two groups were 4.07 ± 2.01 ng/ml and 4.57 ± 2.79 ng/ml, respectively. The results of the analyses revealed that the two groups were significantly different in terms of mean MMP-2 levels (*P* < 0.05). However, as regards the mean TIMP-1 level and MMP-2/TIMP-1 ratio, no significant differences were observed between the sensitive and the resistant groups (*P* > 0.05) (Fig. 3).

## Discussion

*H. pylori* infection is among the leading causes of PUD. Research has revealed that eradicating *H. pylori* in the affected patients not only cures PUD but also improves the related clinical symptoms. PUD heals following the formation of granulation tissue and repair of the injured tissue in the base of the ulcer. The balance between MMPs and TIMPs affects peptic ulcer formation and healing processes. A higher proportion of MMPs increases proteolysis in ECM and causes ulcers, while a higher proportion of TIMPs brings about better protection for ECM, decreases proteolysis, and heals the ulcer [20, 21].

The few studies on the effects of MMP-2 and TIMP-1 on patients with *H. pylori*-induced PUD show controversial results. In 2004, Calabrò et al. conducted a study investigating the variations of MMP-2, MMP-3, MMP-13, and their inhibitor (TIMP-1) in a mouse model with an acetic acid-induced ulcer. They observed that the variations in the levels of MMPs and TIMP-1 were similar during the healing process of the ulcer; their levels increased rapidly after the injury occurrence, reached the highest after one week, and constantly reduced through the last phase of tissue repair. However, quantitative analyses indicated a relative increase in the expression of TIMP-1 in comparison with MMPs during the first week, while after four weeks, the expression of MMPs and TIMP-1 decreased relatively [22]. Our study assessed MMP-2 and TIMP-1 levels and their ratio only once, and the results revealed a higher serum level of MMP-2, TIMP-1, and their ratio, despite all the controversies, which might be due to the higher rate of ECM degradation and the healing process in the presence of multiple ulcers. Furthermore, the presentation of the highest levels of MMP-2 and MMP-2/TIMP-1 ratio, along with the lowest levels of TIMP-1 in the coincidence of gastric and duodenal ulcer, raise the presumption of more severe infection in these patients.

In a study by Rautelin et al., the increase of MMPs in response to *H. pylori*-induced gastritis was investigated. MMP-2, MMP-7, MMP-8, MMP-9, NE, MPO, and TIMP-1 levels were examined. The results indicated that in *H. pylori*-positive patients with gastritis, the serum levels of myeloperoxidase, neutrophil elastase, MMP-8, and MMP-9 increased significantly. Also, MMP-2 and TIMP-1 levels were decreased significantly compared to controls with negative *H. pylori* infection. As regards the serum level of MMP-7, the two groups revealed no significant difference [14]. Their study's sample size was smaller than ours (44 vs. 95 patients, respectively), suggesting the lower precision of the results obtained in their study. Contrary to the findings reported in the mentioned study, our study indicated that MMP-2 and TIMP-1 levels and MMP-2/TIMP-1 ratio significantly increased in patients with *H. pylori*-induced PUD. Studies show higher levels of TIMP-1 in *H.pylori* infection-induced PUD than other reasons, such as NSAIDs [20]. These findings prevail the efficacy of these factors’ measurements in patients with PUD for differentiating the cause of PUDs.

Several studies have been conducted on the effect of alcohol on *H. pylori* infection; some present a negative impact, while others show no significance [23, 24]. But theories such as its antibacterial and bactericidal effects against new and old infections have been proposed for its negative effects. In the types of alcohol that cause stomach acid secretion, it is presumed that as a basal pH is needed for the infection to live in the stomach, the reduction in the stomach pH levels causes this infection to be annihilated [23, 25]. Additionally, alcohol has been found to decrease the activity of MMP-2 in the human body [26]. Our study showed that most of our *H. pylori*-induced PUD patients and most refractory cases did not use alcohol. As the number of alcohol consumers is limited, the significant decrease in MMP-2 levels and less alcohol use in the resistant population does not seem reliable. However, further study on this subject with more population in both groups is suggested.

Yu et al. conducted a review article studying the impact of smoking on H. pylori eradication. They found that smoking affects H. pylori eradication through four mechanisms. Firstly, smokers have increased secretion of pentapeptides. Secondly, nicotine reduces blood flow, resulting in decreased antibiotic delivery. Thirdly, there are changes in proton pump inhibitors metabolism. Fourthly, smoking is linked to lower treatment adherence. Furthermore, smokers with H. pylori-induced PUD have a higher risk of treatment failure [27]. Furthermore, previous studies present a delayed healing of peptic ulcers in smokers through the reduction of gastric blood flow and angiogenesis at the ulcer margin [28]. Our research found no significant correlation between smoking and H. pylori resistance. However, we did discover that MMP-2 levels were considerably higher in smokers, which aligns with the findings of the Ning et al. study [29]. Smoking is well-known as a common but not independent risk factor for PUD [30]. Since MMP-2 levels are elevated in these patients, it is reasonable to assume that smoking may contribute to PUD development in H. pylori patients by increasing MMP-2 levels. We will leave the investigation of this subject to future studies.

In our study, the levels of MMP-2, TIMP-1, and their ratio have been investigated in refractory *H. pylori*-induced PUD for the first time. It is demonstrated that there is a significant increase in the MMP-2 level, but the TIMP-1 level and MMP-2/TIMP-1 ratio both show insignificant results. Based on the existing data, higher levels of MMP-2 in the absence of other diseases can be used as a predictive factor for the existence of refractory *H. pylori*-induced PUD. However, the MMP-2/TIMP-1 ratio might not be used as a refractory *H. pylori*-induced PUD marker.

One of this study’s limitations is that we only focused on serum levels of MMP-2 and TIMP-1 due to our attempts to clarify the inconsistency in their levels in *H. pylori*-induced PUD. However, our findings suggest that these factors may have the potential to predict the resistance of H. pylori infection to treatment, which has not been previously explored.

Future studies should focus on comparing endoscopy-based histopathologic findings and serum levels of these molecules. This can be done by taking deeper biopsies of PUD sites and normal sites, comparing the levels of MMPs and TIMPs between different types and locations of ulcers, and studying their association with the duration of symptoms, as previous studies in mouse models have shown [22]. Additionally, it is recommended to conduct further studies to discover the relationship between other MMPs and TIMPs and the treatment response of H. pylori infection.

## Conclusion

*H. pylori*-induced PUD can increase the MMP-2/TIMP-1 ratio but might not be a good predictor of refractory *H. pylori*-induced PUD. However, the increased MMP-2 level in the absence of other diseases can be used as a predictive marker for refractory *H. pylori*-induced PUD.

## Data Availability

The datasets used and/or analyzed during the current study are available from the corresponding author upon reasonable request.

## Abbreviations

H. pylori: Helicobacter pylori
MMP: Matrix metalloproteinase
TIMP: Tissue inhibitor of metalloproteinase
PUD: Peptic ulcer disease
NSAIDs: Non-steroidal anti-inflammatory drugs
BM: Basement membrane
ECM: Extracellular matrix
ELISA: Enzyme-linked immunosorbent assay
MPO: Myeloperoxidase
NE: Neutrophil elastase
